# Genome-Wide Copy Number Variant Analysis in Inbred Chickens Lines With Different Susceptibility to Marek’s Disease

**DOI:** 10.1534/g3.112.005132

**Published:** 2013-02-01

**Authors:** Juan Luo, Ying Yu, Apratim Mitra, Shuang Chang, Huanmin Zhang, George Liu, Ning Yang, Jiuzhou Song

**Affiliations:** * Department of Animal & Avian Sciences, University of Maryland, College Park, Maryland 20742.; † United States Department of Agriculture-Agriculture Research Service (ARS), Avian Disease and Oncology Laboratory, East Lansing, Michigan 48823; ‡ Bovine Functional Genomic Laboratory, Animal and Natural Resources Institute, United States Department of Agriculture-Agricultural Research Service, Beltsville, Maryland 20705; § Department of Animal Breeding and Genetics, College of Animal Sciences, China Agricultural University, Beijing, 100193, People's Republic of China

**Keywords:** CNV, disease resistance, Marek’s disease, chicken

## Abstract

Breeding of genetically resistant chickens to Marek’s disease (MD) is a vital strategy to poultry health. To find the markers underlying the genetic resistance to MD, copy number variation (CNV) was examined in inbred MD-resistant and -susceptible chicken lines. A total of 45 CNVs were found in four lines of chickens, and 28 were potentially involved in immune response and cell proliferation, etc. Importantly, two CNVs related with MD resistance were transmitted to descendent recombinant congenic lines that differ in susceptibility to MD. Our findings may lead to better strategies for genetic improvement of disease resistance in poultry.

Marek’s disease (MD) is a worldwide problem for poultry industry, with annual loss reports of more than $1 billion (Davison and Nair 2004). MD is caused by Marek’s disease virus (MDV), which is an alphaherpesvirus belonging to the *Mardivirus* genus. There are three serotypes of MDV: serotype 1 (MDV-1), serotype 2 (MDV-2), and serotype 3 [HVT (Davison 2002; Lee *et al.* 2000; Tulman *et al.* 2000)]; however, only MDV-1 is pathogenic and causes a serious lymphoproliferative disease in susceptible chickens. MDV infection in host cells goes through a complex life cycle of multiple phases, defined as early cytolytic, latent, late cytolytic, and transformation phases (Calnek 1986, 2001). Currently, the control of MD mainly relays on vaccination. However, the vaccination efficacy has been experiencing erosion due to the development of the disease itself and emerging new strains of MDV with escalated virulence (Osterrieder *et al.* 2006). Therefore, improving genetic resistance to MD in chickens is a vital approach to augment current control measures.

Two highly inbred lines of chickens (lines 6_3_ and 7_2_, or L6_3_ and L7_2_) were reported to have different susceptibility to MD (Bacon *et al.* 2000), which were used to develop a series of recombinant congenic strains (RCSs) with varied susceptibility to MD (Bacon *et al.* 2000; Silva *et al.* 1996) and different responses to vaccination (Chang *et al.* 2010, 2011). The generation of the RCSs includes one cross between the L6_3_ and L7_2_, two backcrosses of the descendents to L6_3_, followed by full-sib mating. Theoretically, each of the 19 RCSs on average contains approximately 87.5% of the L6_3_ and 12.5% of the L7_2_ genome. Until now, microsatellite markers were used to fingerprint the RCSs (Bacon *et al.* 2000). However, genetic and genomic variations potentially underlying the varied susceptibility to MD in these lines of chickens remain poorly understood.

Single-nucleotide polymorphisms, insertion/deletion polymorphisms, and copy number variations (CNVs) are the major sources of genetic and genomic structural variations in plants, animals, and human (Freeman *et al.* 2006). CNVs are defined as large DNA fragments with sizes ranging from 1 kb to several megabases deleted, inserted, duplicated, or translocated in genome (Beckmann *et al.* 2007). *De novo* and transmitted CNVs are found being involved in a number of diseases, including Crohn’s disease [with a lower copy number of the *DEFB4* gene in humans (Fellermann *et al.* 2006)] and autistic spectrum disorder (Levy *et al.* 2011; Sebat *et al.* 2007). Notably, CNVs also are found to be related with gastrointestinal nematodes resistance and susceptibility in bovine (Hou *et al.* 2011).

In this study, we hypothesized that some CNVs in chicken contribute to MD resistance whereas others to MD susceptibility. Using two highly inbred lines of White Leghorn and two RCSs of the two inbred lines, which vary in resistance/susceptibility to MD, we performed an array comparative genomic hybridization CGH (aCGH) analysis of the four lines of chickens to test our hypothesis. To this end, we identified 45 CNVs in total by comparison among the four lines of chickens. The functions of genes located in CNVs were evaluated for their potential role MD resistance/susceptibility. Finally, we also compared our CNVs with the MD-related quantitative trait loci (QTL) regions.

## Material and Methods

### Animals

A total of six chickens were taken from L6_3_, L7_2_, RCS-L, and RCS-M, which are two recombinant congenic strains from L6_3_ and L7_2_ as mentioned above. The numbers of chickens sampled for this study from the lines were 2, 2, 1, and 1, respectively. The L6_3_ and RCS-L are known resistant to MD, and the L7_2_ and RCS-M are susceptible to MD (Bacon *et al.* 2000). The susceptibility of RCS-M is about half-way between the progenitor L6_3_ and L7_2_. RCS-L is one of most MD-resistant lines out of the RCS series, comparable with the background line L6_3_ (H. M. Zhang *et al.*, 2010, unpublished data). All of the chickens were kept in an Avian Disease and Oncology Laboratory (ADOL)-specific, pathogen-free facility until the bleeding at 15 month of age. All animals were handled closely following the United States Department of Agriculture, Agricultural Research Service, ADOL’s Guidelines for Animal Care and Use (revised April 2005) and the Guide for the Care and Use of Laboratory Animals published by Institute for Laboratory Animal Research (ILAR Guide) in 1996 (http://www.nap.edu/openbook.php?record_id=5140).

### Sample labeling and aCGH analysis

All test genomic DNAs were labeled with Cy3, which were cohybridized with the reference sample that is labeled with Cy5. The whole-genome tiling arrays galGal_WG_CGH, which contain 385k 50-75mer probes, were used to perform the aCGH analysis. The mean and median probe spacing for the array are 2557 bp and 2585 bp, respectively. The hybridization, normalization, and segmentation analysis were performed by NimbleGen Systems Inc. (Madison, WI). A detailed description of their technical specifics can be found on http://www.nimblegen.com/products/lit/lit.html. The calling of candidate CNVs was done the same as before (Liu *et al.* 2011). Briefly, the segments with five or more probes having a mean log2 ratio greater than ± 0.5 (0.5_5) were chosen as candidate CNVs.

### DNA extraction and validation of CNVs by quantitative real-time polymerase chain reaction (PCR)

Genomic DNA from 20 μL of red blood cells was extracted using the DNeasy Blood & Tissue Mini Kit (QIAGEN). Primers for CNVs validation by quantitative real-time PCR were designed based on the probe information using Primer3.0 online primers designer system (http://frodo.wi.mit.edu/) and are shown in Supporting Information, Table S1. Quantitative real-time PCR was performed on the iCycler iQ PCR system (Bio-Rad) in a final volume of 20 µL containing 10 ng of genomic DNA using QuantiTect SYBR Green PCR Kit (QIAGEN) with following procedures: denatured at 95° for 15 min, followed by 40 cycles at 95° for 30 sec, 55−60° for 30 sec, 72° for 30 sec, then extended at 72° for 10 min. For each chicken line, three individuals were used to do the validation. The Red Jungle Fowl (RJF) DNAs were used as the reference which is the same as in the array CGH. The single-copy gene *VIM*, *i.e.*, vimentin (Zehner and Paterson 1983) with primers of forward: 5′-CAGCCACAGAGTAGGGTAGTC-3′; reverse: 5′-GAATAGGGAAGAACAGGAAAT-3′ was used to normalize the amount of input DNA. The Ct value of each test chicken was normalized to the reference gene first, then the ΔCt value was calculated between the test sample and the reference sample (RJF). The relative copy number was calculated as 2^(1- ΔΔCt)^ by assuming that there are two copies of DNAs in the reference region. A linear regression model was used to compare the q-PCR result with the aCGH result.

### RNA extraction and quantitative real-time RT-PCR

RNA was extracted from 20∼30 mg spleen by RNeasy Mini Kit (QIAGEN) and reverse transcribed by QuantiTect Rev. Transcription Kit (QIAGEN). The primers for *ENSGALG00000015816* quantitative real-time PCR are as follows: forward: TTGGACGGGACCTTACAGAC; reverse: TCAGCCTGCAGGAGTGTAAA. The iCycler iQ PCR system (Bio-Rad) and QuantiTect SYBR Green PCR Kit (QIAGEN) were used to do the quantitative PCR to check the expression of *ENSGALG00000015816*. The with following procedures was run on the PCR system: denatured at 95° for 15 min, followed by 40 cycles at 95° for 30 sec, 55−60° for 30 sec, 72° for 30 sec, then extended at 72° for 10 min. The housekeeping gene *GAPDH* (forward: GAGGGTAGTGAAGGCTGCTG; reverse: ACCAGGAAACAAGCTTGACG) was used to normalize the loading amount of cDNA.

### Gene content of the CNVs and Gene Ontology (GO) term analysis

The gene content of the CNVs was obtained by using the UCSC database (Karolchik *et al.* 2004). The Ensembl genes that overlapped with CNVs were extracted. The GO term accession and GO term name information for each Ensembl gene were obtained from BioMart in Ensembl (http://www.ensembl.org/biomart/martview). The enriched GO term was tested by using the hypergeometric distribution. Given a set of genes (*N*), *m* of them are attributed with a particular GO term, then the probability of *k* or more genes from the target gene set (*n*) with this GO term is as follows:

$$P\left(X\;\geq \;k\right)=\sum_{X=k}^{\text{min}\left(m,n\right)}\frac{\left(\begin{array}{c} m\\ k \end{array}\right)\left(\begin{array}{c} N-m\\ n-k \end{array}\right)}{\left(\begin{array}{c} N\\ n \end{array}\right)}$$

### CNVs and QTL overlapping

The MD-related QTL information was downloaded from Chicken QTLdb (http://www.animalgenome.org/cgi-bin/QTLdb/GG/index), which converted the QTL positions from genetic distance (cM) to the physical distance (bp) by using the mapping data from previous research (Heifetz *et al.* 2007, 2009; Liu *et al.* 2001; Vallejo *et al.* 1998). When there is more than 100 bp overlap between CNVs and QTL, we consider that region as shared region. However, as the QTL regions are very big, all the CNVs that we found overlapped with QTL are contained in the QTL region.

### Data releasing

The raw aCGH data have been deposited with the GenBank Gene Expression Omnibus (GEO, http://www.ncbi.nlm.nih.gov/geo/) database under the accession no. GSE38689.

## Results

### Identification of CNVs in chickens with different susceptibility to MD

By using the accepted criteria of 0.5_5 to identify CNVs, we identified a total of 72 CNVs (Table S2) in four chicken lines. We found more CNVs shared between two individuals from the same line than those from two different lines. The percentage of shared CNVs between two individuals was 34.7% and 50% in L6_3_ and L7_2_, respectively; however, there were only 21.1% shared CNVs between L6_3_ and L7_2_. To avoid redundant CNVs, the 72 CNVs were merged into 45 CNVs, which span 3,297,038 bp in length of the chicken genome in four lines of chickens (Table 1 and Table S3). Among the CNVs, 33 (1,921,022 bp) were located on specific chromosome regions and 12 (1,376,016 bp) on uncharacterized chromosome regions (ChrAll_random). However, as with chrUnAll-containing sequences that lack mapping information to the genome, we should handle CNVs on chrUnAll with caution. We found a greater frequency of losses than gains (losses/gains: 31/14). The lengths of the CNVs were ranging from 9950 bp to 387,500 bp with a mean and median of 73,268 bp and 5000 bp, respectively. Because L6_3_ and L7_2_ are highly inbred lines and are also the progenitor lines of RCS-L and RCS-M, we found four shared CNVs with a total length of 769,202 bp among the lines (Table 1). Interestingly, these CNV regions overlap with some genes related with immune system. For example, several genes located in the CNVR deleted on Chr16, including *ENSGALT00000004115* and *ENSGALT00000028239*, are major histocompatibility complex (MHC) class I antigen family genes.

**Table 1:** Distribution of CNVs in different chicken lines

Sample	CNV Count	Gain	Loss	Total Length, bp	Shared (Gain/Loss)	Shared Length, bp	Unique (Gain/Loss)	Unique Length, bp
L6_3_	22 (11)	8 (4)	14 (7)	1,915,598			6 (1/5)	300,059
L7_2_	20 (10)	5 (2.5)	15 (7.5)	1,790,92	4 (2/2)	769,202	12 (3/9)	869,721
RCS-L	12 (12)	4 (4)	8 (8)	1,390,273			1 (0/1)	15,000
RCS-M	21 (21)	8 (8)	13 (13)	2,032,661			9 (3/6)	413,217

The numbers in parentheses from columns 2 to 4 are CNV events per individual. The numbers in parentheses in column 6 and 8 are the gain/loss ratio of CNVs. Shared: CNVs shown in all four chicken lines; Unique: CNVs shown only in one chicken line. CNV, copy number variation.

### Validation of CNVs

**Figure 1:**
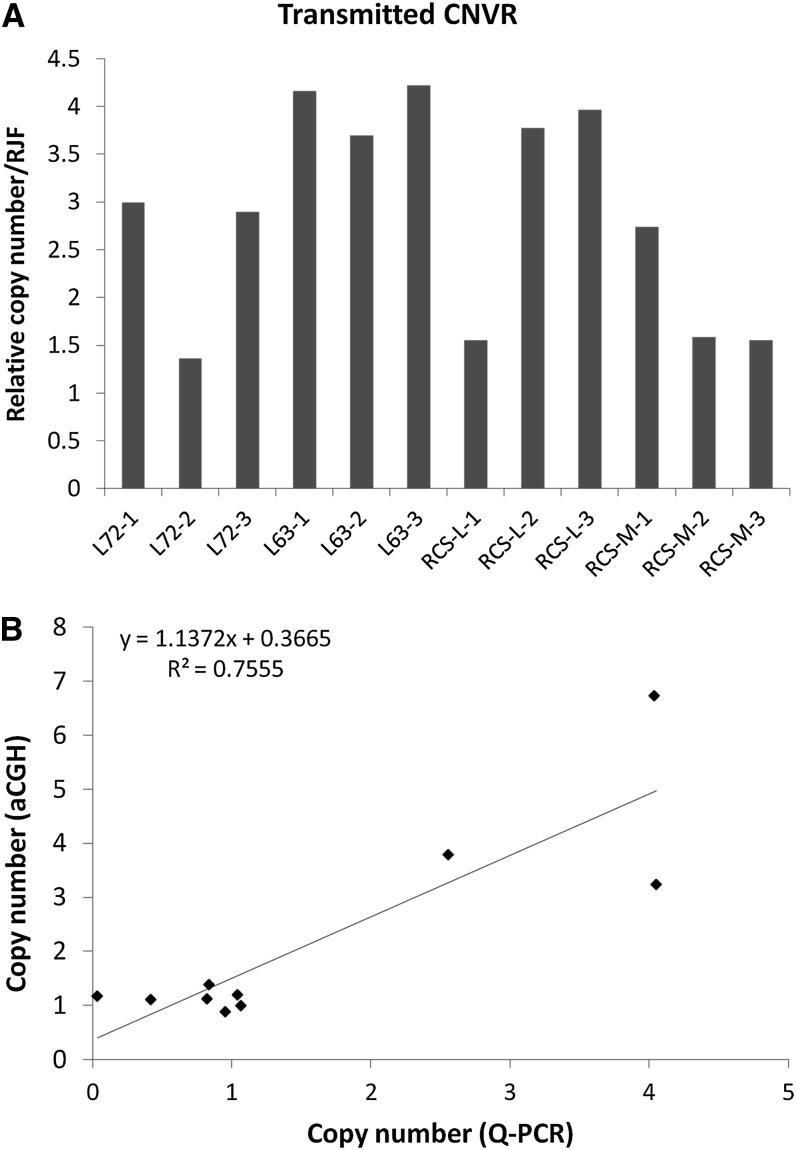
Validation of copy number variations by Q-PCR. (A) Q-PCR validation of a region (Chr2:49165364-49227941) showed transmitted CNV from L6_3_ and L7_2_ to RCS-L and RCS-M which was gain in all four chicken lines. (B) Regression analysis of the copy number estimated from aCGH and qPCR, which show a linear relationship between them.

Before validating the CNVs, we compared our result with the published CNVs identified in White Leghorn chickens (Griffin *et al.* 2008; Wang *et al.* 2010). We found seven CNVs that overlapped with the reported CNVs (Table S4). The length of the overlap between these two sets of CNVs ranged from 17,814 bp to 207,851 bp. Then, a quantitative (Q)-PCR method was performed on selected CNVs, including one region (Chr2:49165364-49227941) found in all four chicken lines, which is considered as a transmitted CNV from L6_3_ and L7_2_ to RCS-L and RCS-M. Compared with RJF, most of the L6_3_, L7_2_, RCS-M, and RCS-L chickens tested had more than two copies in this region although several individuals showed different results, especially in RCS-M (Figure 1A). Then, 10 other CNVs were randomly chosen to for Q-PCR analysis. The regression analysis showed a strong linear correlation between the copy number identified by both Q-PCR and aCGH (Figure 1B).

### Transmitted CNVs

Theoretically, for RCS-L and RCS-M, each on average should have a random sample of approximately 87.5% of the background L6_3_ genome and 12.5% of the donor L7_2_ genome, respectively. We found that approximately 91.7% and 66.7% of the CNVs in RCS-L and RCS-M were inherited from the progenitor lines L6_3_ and L7_2_, respectively (Table 2). The absolute numbers of the inherited CNVs in the two descendent lines were similar. There were seven CNVs that were shared between the L6_3_ and L7_2_, most of which were transmitted to the descendent RCS-L (6 of 7) and RCS-M (5 of 7) chickens. Approximately one-half of the CNVs that were unique for L6_3_ were transmitted to RCS-L (7 of 15) and RCS-M (8 of 15). However, the CNVs that were unique for L7_2_ were rarely transmitted: none to RCS-L and only one to RCS-M. In terms of the length of transmitted CNVs, approximately 92.7% and 88.6% of the CNV sequences in RCS-L and RCS-M were from L6_3_, respectively, which is close to the theoretical expectation of 87.5%. Except for the transmitted CNVs there are also one CNVs in RCS-L and seven in RCS-M that are not shown in the progenitor chicken lines, which could be the potential lineage-specific CNVs.

**Table 2:** Transmitted CNVs from L6_3_ and L7_2_ in RCS-L and RCS-M

Sample	Total Count	Transmitted Count	From Both L6_3_ and L7_2_	Length, bp	L6_3_ Only	Length L6_3_, bp	L7_2_ only	Length L7_2_, bp
RCS-L	12	11 (4/7)	6 (2/4)	798,799	7 (4/3)	576,474	0 (0/0)	0
RCS-M	21	14 (7/7)	5 (2/3)	807,972	8 (5/3)	727,970	1 (0/1)	83,502

The numbers in parentheses are the gain/loss ratio. CNV, copy number variation; RCS, recombinant congenic strain.

### Comparison of the CNVs between the resistant and susceptible chicken lines

**Figure 2:**
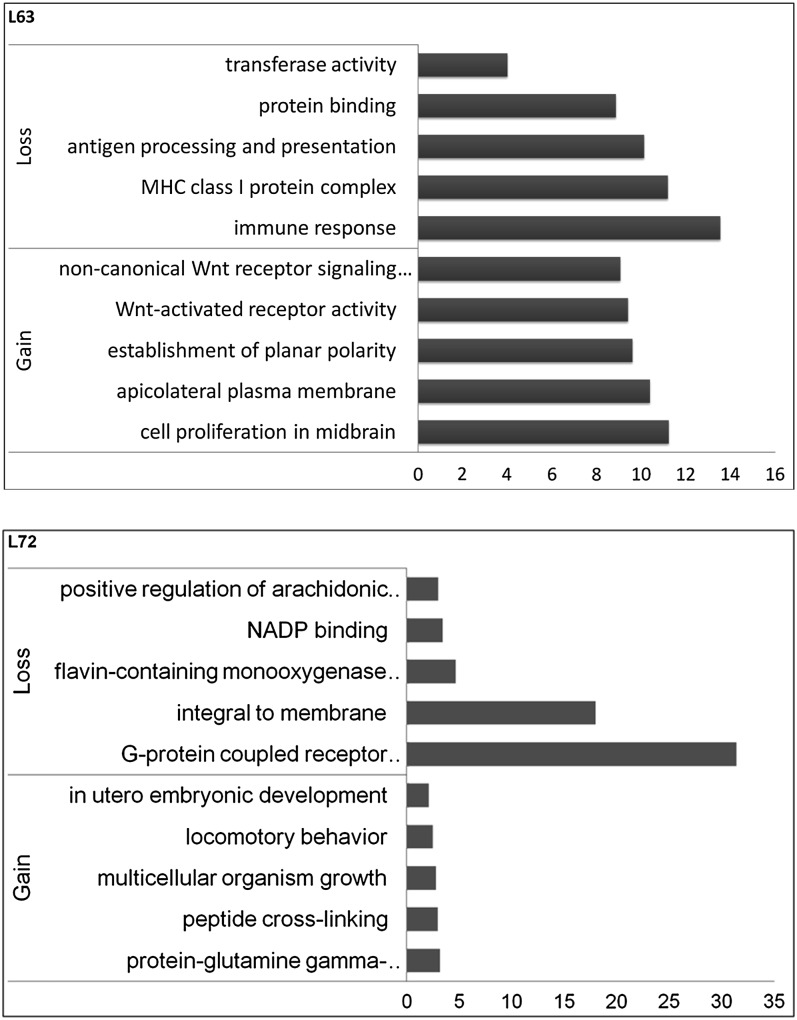
GO term analysis of differentially presented CNVs between L6_3_ and L7_2_. The x-axis shows the –Log(P value) of the enriched GO term.

Specifically, all genomic variations between the L6_3_ and L7_2_ could be considered as candidate regions for MD-resistance or -susceptibility. By comparison, we found that a total of 28 CNVs spanning 2,031,252 bp differed between the two lines of chickens (Table S3). These 28 CNVs encompass several functional genes, including AP2-associated kinase 1(*ENSGALT00000000026*), amyloid beta (A4) precursor protein-binding, family A, and member 2 (*ENSGALT00000006262*). GO term analysis was then used to find the functions of the genes located in the differentially presented CNVs (Figure 2). For L6_3_ unique CNVs, the deleted sequences contained genes that are related to immune response, MHC class I protein complex, antigen procession, and presentation; the duplicated genes have functions of cell proliferation in midbrain, establishment of planar polarity, and Wnt-activated receptor activity. For the L7_2_ unique CNVs, the lost genes are enriched in G-protein coupled receptor signaling pathway, flavin-containing monooxygenase activity, and NADP binding; the genes duplicated have functions of protein-glutamine gamma-glutamyltransferase activity, peptide cross-linking, and multicellular organism growth. As shown previously, we found many transmitted CNVs in RCS-L and RCS-M that have different phenotypes after MDV infection. The resistant CNVs and susceptible CNVs candidate that are transmitted to RCS-L and RCS-M respectively can be considered high confident CNVs for MD-resistance and MD-susceptibility. Thereafter, we found one MD-resistant candidate CNV that is a loss of genomic region on chromosome 19 spanning 50 kb and one MD-susceptible candidate CNV that is also a loss of genomic region on uncharacterized chromosomes region spanning 83.5kb. One functional gene was found located on the MD-resistant candidate CNV, which is the general transcription factor IIi (*GTF2I*). However, only several ESTs were found in the MD-susceptible CNV, but no known functional genes were identified.

### CNVs and gene expression

**Figure 3:**
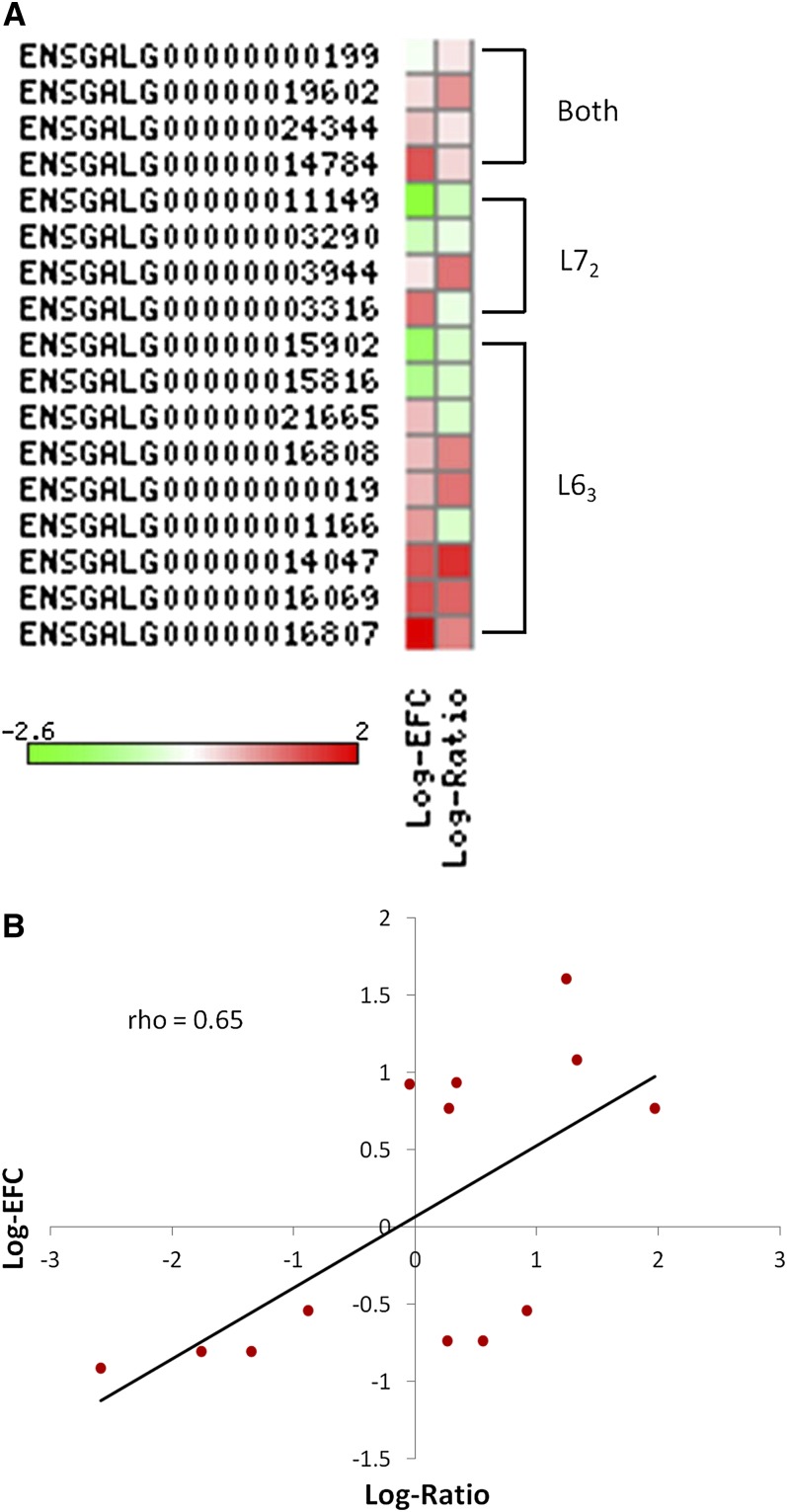
Relationship between CNVs and gene expression. (A) Heatmap representation of the relationship between the gene located in CNVs and their expression. Both: the CNVs shown in both L6_3_ and L7_2_; Log-EFC: log expression fold change between L6_3_ and L7_2_; log-ratio: log raw signal ratio between L6_3_ or L7_2_ and Red Jungle Fowl, which represent the CNVs. Green indicates that the expression is down-regulated or the CNV is lost; Red indicates that the expression is up-regulated or the CNV is gained. (B) Positive correlation between gene copy number differences and gene expression differences in CNVs that shown differences between L6_3_ and L7_2_.

**Figure 4:**
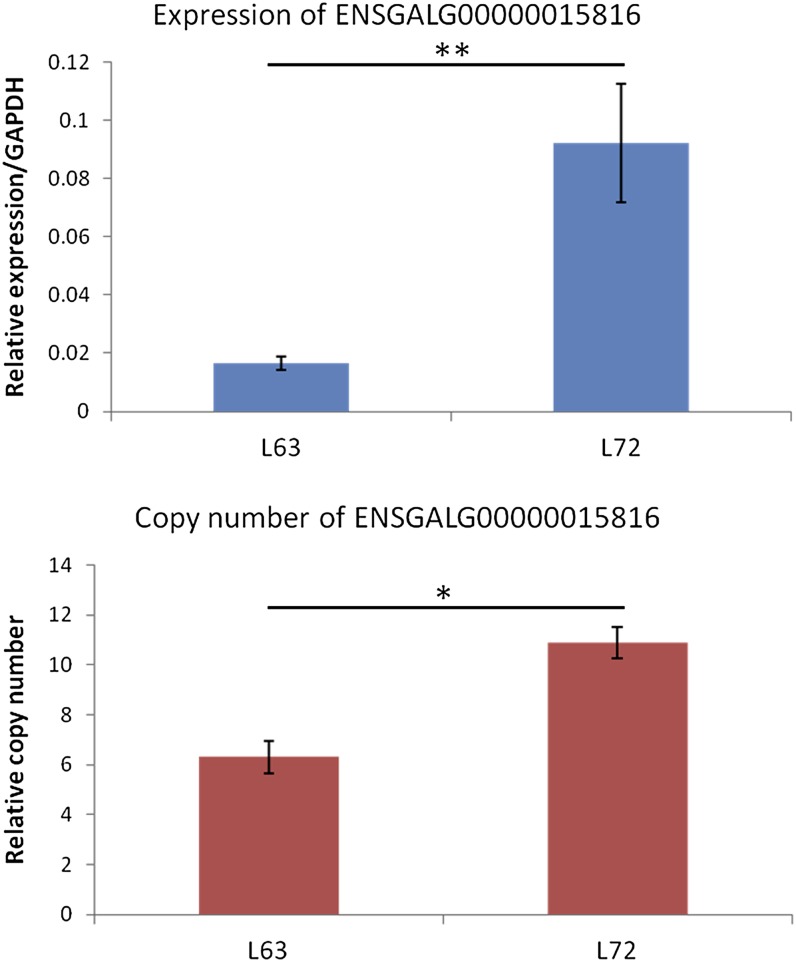
Validation of the positive correlation between CNV and gene expression level in our population. The copy number (bottom) and gene expression level (top) were tested in the same individuals. *P < 0.05, **P < 0.01, N = 4.

CNVs overlapped with genes were found regulating the gene expression (Stranger *et al.* 2007). To check the relationship between CNVs and gene expression in our CNVs, we extracted expression information from our previous microarray data (Yu *et al.* 2011) for genes located in the CNVs. In the microarray trial, we used the exact same chicken lines, L6_3_ and L7_2_, to perform gene expression analysis in chicken spleens in a MDV challenge experiment to check whether the host responses to the virus infection are different in chickens with different susceptibility to MD. The log-fold change of the differential gene expression between L6_3_ and L7_2_ was used for the following correlation analysis between gene expression and CNVs. In addition, the log-ratio of the signal density between L6_3_ and L7_2_ also was calculated from the raw data to show the different copy numbers between L6_3_ and L7_2_. For the CNVs that were shared in both L6_3_ and L7_2_ chickens, the expression of the genes in them are similar between these two chicken lines (Figure 3A). The expression of encompassed genes didn’t differ significantly between the two lines, and the correlation coefficient (rho = 0.09) was negligible (Figure S1). However, for unique CNVs, the gene expression differed significantly between L6_3_ and L7_2_. For most of genes, gain/loss of the copy number is related with the up-/down-regulation of the expression level (Figure 3A). The Pearson correlation analysis showed a high positive correlation (rho = 0.65) between the copy number and the gene expression for line-specific CNVs (Figure 3B). To further confirm the positive correlation of CNV and gene expression in our population, we picked up a candidate gene (ENSGALG00000015816) from the upper list and analyzed the copy number and gene expression level in our population. As shown in Figure 4, when the copy numbers of the candidate gene are significantly greater in L7_2_ chickens, the expression is significantly greater.

### CNVs and MD-related QTL

It is reported that the QTL for MD resistance and susceptibility were mapped to several chromosomes, including GGA 1-5, 7-9, 15, 18, 26, Z, E21, and E16, by using the backcross or intercross populations (Heifetz *et al.* 2007, 2009; Liu *et al.* 2001; Vallejo *et al.* 1998). To identify whether common genomic variants were detected by using different methods, we compared our CNVs with the QTL regions. We found that those QTL regions overlapped with one of the shared CNVs, five CNVs only found in the resistant line, two CNVs only found in the susceptible line, and three of line-specific CNVs in RCS-M (Table 3). These overlapped CNVs were located on GGA 1, 2, 5, 9, 15, and Z, spanning 542,614 bp of the chicken genome. Among these overlapped regions, several functional genes were found, including LIM and senescent cell antigen-like domains 1 (*LIMS1*), myosin light chain kinase family, member 4 (*MYLK4*), frizzled family receptor 6 (*FZD6*), and brain and acute leukemia, cytoplasmic (*BAALC*).

**Table 3:** Overlap between QTL and CNVs

QTL Region	Overlapped CNVs Region	CNV Type	Gain/Loss	CNV Length, bp	Genes
Chr1:33590456-195276750	Chr1:48000008-48042918	RCS-M	Loss	42,910	NA
	Chr1:127112500-127162500	L7_2_	Gain	50,000	NA
	Chr1:140556250-140658750	L6_3_	Gain	102,500	LIM and senescent cell antigen-like domains 1 (*LIMS1*)
Chr2:23112333-139609547	Chr2:40660120-40677934	L7_2_	Gain	17814	Chromosome 3 open reading frame 77 (*C3orf77*)
	Chr2:49165364-49227941	Shared	Gain	62,577	Similar to T-cell receptor gamma chain Vg3-Jg1
	Chr2:68752514-68767752	L6_3_	Loss	15,238	Myosin light chain kinase family,member 4 (*MYLK4*)
	Chr2:134725000-134831250	L6_3_	Gain	106,250	Frizzled family receptor 6 (*FZD6*), brain and acute leukemia, cytoplasmic (*BAALC*)
Chr5:3903453-23182732	Chr15:11013750-11031250	L6_3_	Loss	85,284	NA
Chr9:3714402-23441680	Chr9:4962500-4987500	L6_3_	Gain	25,000	NA
Chr15:4923592-12656803	Chr15:11013750-11031250	RCS-M	Loss	17,500	NA
ChrZ:21455210-68018681	ChrZ:66282807-66300348	RCS-M	Loss	17,541	NA

QTL, quantitative trait loci; CNV, copy number variation; RCS-M, *de novo* CNVs in RCS-M; L6_3_, resistant candidate CNVs in L6_3_; L7_2_, susceptible CNVs in L7_2_; Shared, CNVs shared in four chicken lines. NA, not available.

## Discussion

In this study, we studied the CNVs in inbred chicken lines with different susceptibility to MD by aCGH. We found that four shared CNVs among the four chicken lines contain some immune-related genes, most of which are MHC class I antigen family genes. Because we used RJF as our reference to get these CNVs, this result indicates that compared with the wild chicken (RJF), the domesticated chicken lines (White Leghorn) have less copies of the immune related genes, which suggests that domestication may influence the immunity system of the chicken. In the meantime, when compared with reported CNVs identified in White Leghorns (Griffin *et al.* 2008; Wang *et al.* 2010), only seven shared CNVs were found, and most of the CNVs were different in our findings, which indicated different sample, breed, and platform may lead to CNV detection differences. It is also possible that a long term of selection for disease resistant in the lines of chickens led to many lineage-specific events of CNVs.

CNVs are found in relation with disease resistance in both humans and bovine (Gonzalez *et al.* 2005; Hou *et al.* 2011). To evaluate the functions of CNVs in chickens, we examined the CNVs profiles in two highly inbred lines (L6_3_ and L7_2_) and two recombinant congenic strains (RCS-L and RCS-M) derived from them in this study. The rational was that because the susceptibility to MD was known ranking as L7_2_ > RCS-M > RCS-L > L6_3_, the genomic structure variations between RCS-M and RCS-L inherited from the L6_3_ and L7_2_ may account for, in part, the different susceptibility to MD. In our experiment, we indeed detected the CNVs of L6_3_ and L7_2_ shown in the RCS-L and RCS-M genome. Most of CNVs in RCS-L (91.7% of total CNVs) and RCS-M (66.7% of total CNVs) were transmitted from L6_3_ and L7_2_ as expected, and presumably most of CNVs inherited from the L6_3_ may be contributable to MD resistance in RCS-L and RCS-M as compared to CNVs from the L7_2_. The CNVs that were only indentified in the RCS-L and RCS-M are considered as lineage-specific CNVs, which may also explain the phenotypic variations between the two RCSs.

We further explored the potential functionality of the CNVs that are differentially presented between L6_3_ and L7_2_. These CNVs found encompassed genes involved in immune response, the MHC class I protein complex, and antigen processing and presentation. Because the same MHC haplotype B_2_ was found in L6_3_ and L7_2_ chickens (Davison and Nair 2004) and there were no DNA sequence variations of the MHC *B-FIV* identified (Vallejo *et al.* 1998), for a long time the MD resistance in L6_3_ was attributable to non-MHC genomic variation. The CNV identified in this study indicated that MHC may also contribute to the primarily non-MHC−associated resistance but in a CNV manner instead of a single-nucleotide polymorphism or insertion/deletion polymorphism. A similar case also was found in other species, such as rat (Roos and Walter 2005) and Rhesus macaque (Otting *et al.* 2005). Generally speaking, the high level of MHC polymorphism is considered beneficial for disease resistance (Wallny *et al.* 2006). In humans, the lower copy number of MHC is associated with the risk of systemic lupus erythematosus susceptibility (Yang *et al.* 2007). However, in Tasmanian devils, less MHC may help the animals escape from the disease epidemic (Siddle *et al.* 2010). The results in our case are similar to the Tasmanian devils.

The question now is how the CNVs differentially represented between the resistant and susceptible lines of chickens modulate disease resistance or susceptibility. One aspect is clear, that is, the gain or loss of a DNA region would influence the expression of the genes in which the CNVs are located. Some researchers found a complicated relationship between gain or loss of CNVs and gene expression in human (Beckmann *et al.* 2007; Henrichsen *et al.* 2009; Stranger *et al.* 2007). In this study, we found that CNVs were positively correlated with gene expression, indicating that the CNVs may influence the host in MD resistance or susceptibility through regulating the expression of the genes.

In the meantime, to confirm the power of disease resistance breeding that may also be shown in other genomic variants, we overlapped the CNVs with the MD-related QTL regions that were identified in previous study. Of all the candidate MD-resistant and -susceptible CNVs, 25% (7/28) of them are overlapped with the QTL. Five functional gene were found located in these overlapped regions, namely *LIMS1*, *MYLK4*, *C3orf77*, *FZD6*, and *BAALC*. Three of them were reportedly being related with cancers. *LIMS1* was found up-regulated in cancer samples (Wang-Rodriguez *et al.* 2002) and is required for the apoptosis resistance of cancer cells through involving with the ERK-Bim pathway (Chen *et al.* 2008). *BAALC* was found overexpressed in a subset of acute leukemias (Baldus *et al.* 2003; Tanner *et al.* 2001). However, the *FZD6* functions as an inhibitor of cancer cell transformation by negatively regulating canonical Wnt/β-catenin signaling pathway (Golan *et al.* 2004).

In conclusion, by using aCGH, we identified a total of 28 candidate CNVs, which may contribute to MD resistance and/or susceptibility in chickens, within which approximately 25% were overlapped with previous identified MD-related QTL. A positive correlation between CNVs and expression of genes encompassing the CNVs was observed, which suggested that the candidate CNVs may influence the susceptibility to MD through regulation of the gene expression. These findings provided additional information elucidating possible mechanisms underlying genetic resistance to MD, which may help to improve strategies to better control of MD in poultry.

## Supplementary Material

Supporting Information
