# Supplementary material for: Genome-Wide Copy Number Variant Analysis in Inbred Chickens Lines With Different Susceptibility to Marek’s Disease
Source: G3 (Bethesda). 2013 Feb 1;3(2):217–23. doi: 10.1534/g3.112.005132 (PMC3564982; doi:10.1534/g3.112.005132)
Supplement: Supporting Information [file supp_3_2_217__index.html]

Supporting Information 

# Genome-Wide Copy Number Variant Analysis in Inbred Chickens Lines With Different Susceptibility to Marek’s Disease

## Supporting Information for Luo *et al.*, 2013

**Files in this Data Supplement:**

- Supporting Information - Figure S1 and Tables S1-S4 (PDF, 171 KB)
- Figure S1 - Correlation analysis between the gene copy number changes and gene expression changes (PDF, 86 KB)
- Table S1 - Primer for Q-PCR validation of CNVRs (PDF, 56 KB)
- Table S4 - Comparison of the CNVRs between previous finding and our current finding (PDF, 47 KB)
- Table S2 - CNVs identified in 6 chickens from 4 chicken lines (.xlsx, 13 KB)
- Table S3 - Merged CNVRs (.xlsx, 10 KB)
