# Supplementary material for: Genome-Wide Copy Number Variant Analysis in Inbred Chickens Lines With Different Susceptibility to Marek’s Disease
Source: G3 (Bethesda). 2013 Feb 1;3(2):217–23. doi: 10.1534/g3.112.005132 (PMC3564982; doi:10.1534/g3.112.005132)
Supplement: Supporting Information [file supp_3.2.217_005132SI.pdf]

G3 · Genes | Genomes | Genetics

Genome-wide copy number variant analysis in inbred chickens lines with different susceptibility to Marek's disease

Juan Luo[1], Ying Yu[1, 4], Apratim Mitra[1], Shuang Chang[2], Huanmin Zhang[2], George Liu[3], Ning Yang[4], Jiuzhou Song[1§]

[1]Department of Animal & Avian Sciences, University of Maryland, College Park, MD 20742, USA

[2]USDA, ARS, Avian Disease and Oncology Laboratory, East Lansing, MI 48823, USA

[3] Bovine Functional Genomic Laboratory, Animal and Natural Resources Institute, USDA-Agricultural Research Service, Beltsville, MD 20705, USA

[4]Current address: Department of Animal Breeding and Genetics, College of Animal Sciences, China Agricultural University, Beijing, 100193, P.R. China

[§]Corresponding author: songj88@umd.edu

DOI: 10.1534/g3.112.005132

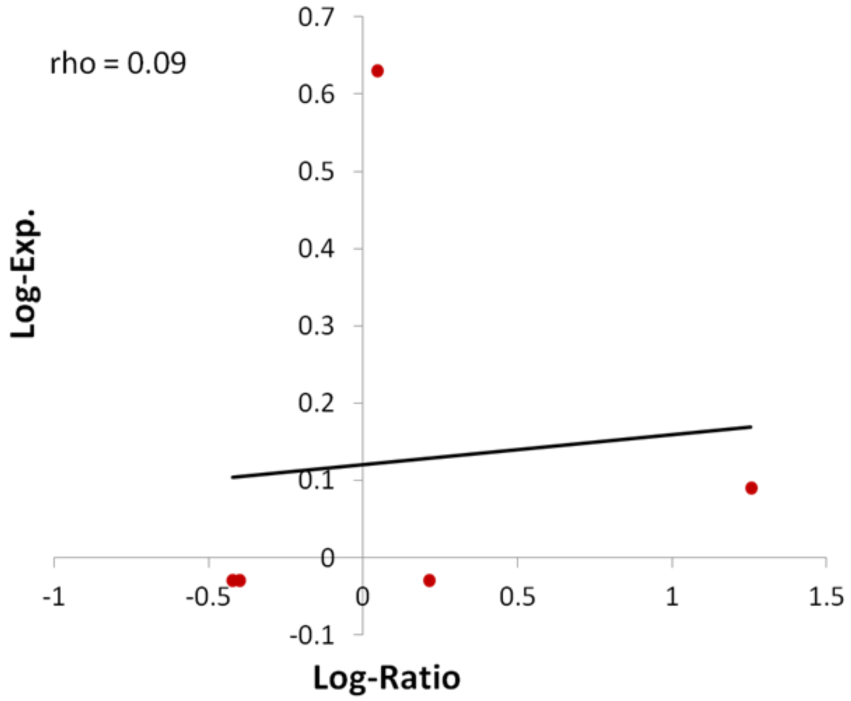

Figure S1 Correlation analysis between the gene copy number changes and gene expression changes. Almost no correlation was identified between the gene copy number and gene expression in the CNVRs that are shared between $L6_3$ and $L7_2$ due to no or very small changes of both.

Table S1 Primer for Q-PCR validation of CNVRs

Primer Name	CNVR Position	Sequences (5'→3')	Product Length (bp)
CNV-1	Chr2:49165364-49227941	Foward:GTTCTGTTCTGGGCTTCTCG<br>Reverse:GCATTGGTAAGATGCCCACT	166
CNV-2	Chr18:2340193-2350143	Foward:GATGAGAGGAACGCCTTGAG<br>Reverse:ATGCAGAACCCAAGGACAAC	131
CNV-3	Chr19:2621250-2666250	Foward:CAACCTGCTCCAGTGTCTCA<br>Reverse:CCTGAAAATGGCCTACAGGA	160
CNV-4	Chr3:35342943-35365200	Foward:ATGACAGGCACATGCAAAAA<br>Reverse:TGCCCAAACAAAATCATTCA	199
CNV-5	Chr11:202-20340	Foward:ATGATCTGGACCTGCTGGAG<br>Reverse:CATGGAGCCACCAAGGTACT	153
CNV-6	Chr7:17756250-17806250	Foward:CGTGAAGGACGGGTTCTAAG<br>Reverse:GAGGAAAGCTACGGATGCAG	173
CNV-7	ChrZ:662828070-66300348	Foward:ACCCAGGCCCTTCTATGATT<br>Reverse:CTTTCCTGCTCAAGCGAAAC	166
CNV-8	Chr3:37136250-37151250	Foward:TCAACTGCTGGTGTGATTGA<br>Reverse:TGAAGTTCAACCCAACACAAA	174
CNV-9	Chr3:17568750-17631250	Foward:CCGTTCTCAGACACGATGAA<br>Reverse:TTGTCAAAACGAGCAACGAG	171
CNV-10	Chr2:40660120-40677934	Foward:CGAGTTTTGAAGCCTTGCTC<br>Reverse:CAAGGTCTGGCTGTTCCAGT	158
CNV-11	Chr10:6842856-6867633	Foward:AATGCTGCTGACAAGCACTG<br>Reverse:CAAGCTTCATCCTGGCTCTC	130

Tables S2 and S3

Available for download at http://www.g3journal.org/lookup/suppl/doi:10.1534/g3.112.005132/-/DC1.

Table S2 CNVs identified in 6 chickens from 4 chicken lines

Table S3 Merged CNVRs

Table S4 Comparison of the CNVRs between previous finding and our current finding

Previous finding								Our current finding						
Wang etal, 2010				Griffin etal, 2008										
Chr.	Start	End	W.L.	Chr.	Start	End	W.L.	Chr.	Start	End	$L6_3$	$L7_2$	RL	RM
2	40647961	40687894	G	-	-	-	-	2	40660120	40677934	-	G	-	-
2	134727846	134830176	G	-	-	-	-	2	134725000	134831250	G	-	G	G
4	88897639	89072982	L	4	88935000	89025000	L	4	88905489	89093750	L	-	L	L
5	22120222	22212778	L	-	-	-	-	5	22117506	22202790	L	-	L	L
16	270019	432851	L	16	15000	426425	L	16	225000	432851	L	L	L	L
-	-	-	-	2	49185000	49215000	G	2	49165364	49227941	G	G	G	G
-	-	-	-	3	11355000	113646334	G	3	113597540	113652668	-	-	-	G

W.L.: White Leghorn; RL: RCS-L; RM: RCS-M; G: gain; L: loss. -: not found.
