# Supplementary material for: Genome-Wide Copy Number Variant Analysis in Inbred Chickens Lines With Different Susceptibility to Marek’s Disease
Source: G3 (Bethesda). 2013 Feb 1;3(2):217–23. doi: 10.1534/g3.112.005132 (PMC3564982; doi:10.1534/g3.112.005132)
Supplement: Supporting Information [file supp_3.2.217_FigureS1.pdf]

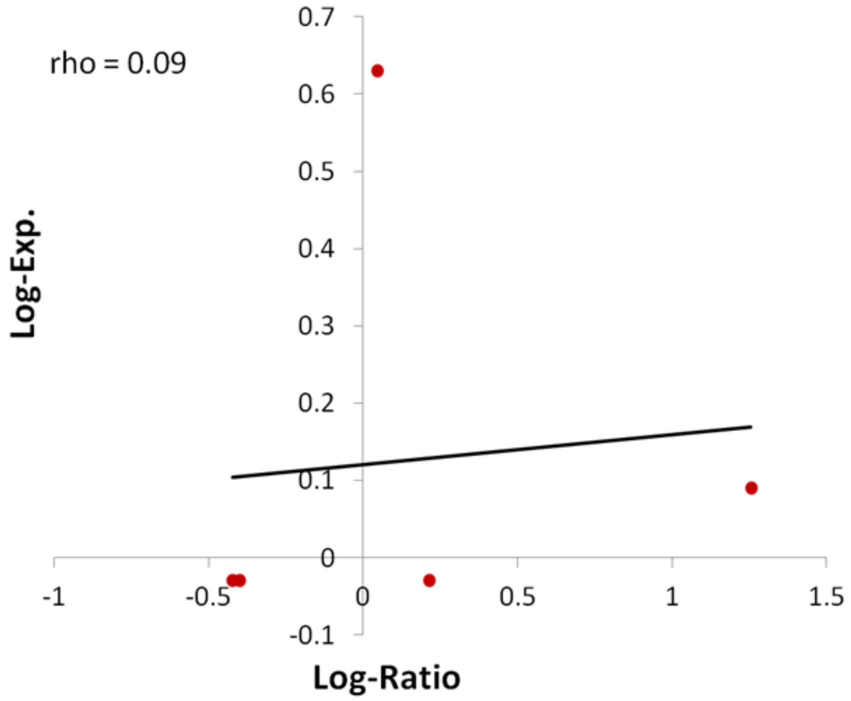

Figure S1 Correlation analysis between the gene copy number changes and gene expression changes. Almost no correlation was identified between the gene copy number and gene expression in the CNVRs that are shared between $L6_3$ and $L7_2$ due to no or very small changes of both.
