# Supplementary material for: Genome-Wide Copy Number Variant Analysis in Inbred Chickens Lines With Different Susceptibility to Marek’s Disease
Source: G3 (Bethesda). 2013 Feb 1;3(2):217–23. doi: 10.1534/g3.112.005132 (PMC3564982; doi:10.1534/g3.112.005132)
Supplement: Supporting Information [file supp_3.2.217_TableS1.pdf]

Table S1 Primer for Q-PCR validation of CNVRs

Primer Name	CNVR Position	Sequences (5'→3')	Product Length (bp)
CNV-1	Chr2:49165364-49227941	Foward:GTTCTGTTCTGGGCTTCTCG<br>Reverse:GCATTGGTAAGATGCCCACT	166
CNV-2	Chr18:2340193-2350143	Foward:GATGAGAGGAACGCCTTGAG<br>Reverse:ATGCAGAACCCAAGGACAAC	131
CNV-3	Chr19:2621250-2666250	Foward:CAACCTGCTCCAGTGTCTCA<br>Reverse:CCTGAAAATGGCCTACAGGA	160
CNV-4	Chr3:35342943-35365200	Foward:ATGACAGGCACATGCAAAAA<br>Reverse:TGCCCAAACAAAATCATTCA	199
CNV-5	Chr11:202-20340	Foward:ATGATCTGGACCTGCTGGAG<br>Reverse:CATGGAGCCACCAAGGTACT	153
CNV-6	Chr7:17756250-17806250	Foward:CGTGAAGGACGGGTTCTAAG<br>Reverse:GAGGAAAGCTACGGATGCAG	173
CNV-7	ChrZ:662828070-66300348	Foward:ACCCAGGCCCTTCTATGATT<br>Reverse:CTTTCCTGCTCAAGCGAAAC	166
CNV-8	Chr3:37136250-37151250	Foward:TCAACTGCTGGTGTGATTGA<br>Reverse:TGAAGTTCAACCCAACACAAA	174
CNV-9	Chr3:17568750-17631250	Foward:CCGTTCTCAGACACGATGAA<br>Reverse:TTGTCAAAACGAGCAACGAG	171
CNV-10	Chr2:40660120-40677934	Foward:CGAGTTTTGAAGCCTTGCTC<br>Reverse:CAAGGTCTGGCTGTTCCAGT	158
CNV-11	Chr10:6842856-6867633	Foward:AATGCTGCTGACAAGCACTG<br>Reverse:CAAGCTTCATCCTGGCTCTC	130
