# Supplementary material for: Genome-Wide Copy Number Variant Analysis in Inbred Chickens Lines With Different Susceptibility to Marek’s Disease
Source: G3 (Bethesda). 2013 Feb 1;3(2):217–23. doi: 10.1534/g3.112.005132 (PMC3564982; doi:10.1534/g3.112.005132)
Supplement: Supporting Information [file supp_3.2.217_TableS4.pdf]

Table S4 Comparison of the CNVRs between previous finding and our current finding

Previous finding								Our current finding						
Wang etal, 2010				Griffin etal, 2008										
Chr.	Start	End	W.L.	Chr.	Start	End	W.L.	Chr.	Start	End	$L6_3$	$L7_2$	RL	RM
2	40647961	40687894	G	-	-	-	-	2	40660120	40677934	-	G	-	-
2	134727846	134830176	G	-	-	-	-	2	134725000	134831250	G	-	G	G
4	88897639	89072982	L	4	88935000	89025000	L	4	88905489	89093750	L	-	L	L
5	22120222	22212778	L	-	-	-	-	5	22117506	22202790	L	-	L	L
16	270019	432851	L	16	15000	426425	L	16	225000	432851	L	L	L	L
-	-	-	-	2	49185000	49215000	G	2	49165364	49227941	G	G	G	G
-	-	-	-	3	11355000	113646334	G	3	113597540	113652668	-	-	-	G

W.L.: White Leghorn; RL: RCS-L; RM: RCS-M; G: gain; L: loss. -: not found.
